# Oral Ulceration With Bone Sequestration: Diagnostic Challenge, Management Strategy

**DOI:** 10.1002/ccr3.70319

**Published:** 2025-04-09

**Authors:** Marc Dieb, Lea Dieb, Claude Robert

**Affiliations:** ^1^ ADORES Dental Selas Paris France; ^2^ ADORES Scientific Research Paris France; ^3^ GLIAXONE Saint‐Germain‐Sous‐Doue France

**Keywords:** case report, dental extraction complications, mylohyoid ridge, necrotic bone management, oral ulceration, post‐extraction ulceration

## Abstract

Involving ulceration over non‐vital bone without osteonecrosis factors, Oral Ulceration with Bone Sequestration (OUBS) is underreported in the scientific and medical literature. We hope that our study will: (i) help health professionals in diagnosing and managing OUBS, (ii) encourage them to publish their experiences, and (iii) inspire researchers to develop models to address unresolved questions about this condition.

Osteonecrosis of the jaw (ONJ) is defined as exposed bone within the oral cavity that fails to heal over 8 weeks after identification. Most reported cases are associated with medication such as bisphosphonates, denosumab, or zoledronic acid, or previous radiotherapy of the head and neck [1]. However, in rare cases, the maxillary or mandibular bone may become exposed, with accompanying necrosis/sequestration and ulceration of the adjacent oral mucosa. This long‐lasting condition that does not fit the conventional definition of ONJ is referred to as Oral Ulceration with Bone Sequestration (OUBS) and requires specific treatment [2].

OUBS develops principally at different sites of osseous prominences covered by thin mucosa, such as the lingual mandibular surface along the mylohyoid ridge, tori, and exostoses [3]. Interestingly, the mylohyoid ridge and other osseous prominence sites of the maxilla are covered with thin connective tissue oral mucosa that makes them susceptible to laceration and ulceration induced by mechanical forces such as mastication or accidental trauma. Additionally, these bony sites are poorly nourished due to their distance from the alveolar blood supply [4]. Secondary bacterial colonization may lead to acute inflammation, characterized by acidity and reduced local oxygen tension, resulting in local ischemia, bone necrosis, and consequently sequestration [2]. As long as the bone sequestrum persists, it maintains the ulceration and delays healing [2].

Clinically, it presents as a painful or non‐painful ulceration with a hard center typically surrounded by swollen, erythematous, and fragile soft tissues. If not swiftly managed, the exposed necrotic bone can entirely sequestrate [4].

Diagnosis is primarily based on the clinical description and the specific nature of the lesion's development site, which reflects its likely pathogenic mechanism. OUBS should only be considered as a diagnosis after excluding other possible causes [2, 3].

Though the term OUBS appeared recently in the medical and scientific literature, it must be noted that this condition was previously documented under various expressions such as “lingual mandibular sequestration and ulceration” (for details see the retrospective study of Thermos et al. [2]). Consequently, literature in which the term “OUBS” is used remains scarce: a search on PubMed database by a group of 34 experts issued from the *International Task Force on Osteonecrosis of the Jaw* made 10 years ago identified only eight papers composed of case reports or case series on oral ulceration and benign sequestration [1]; and this rarity was also stated more recently [2, 3]. Moreover, this deficiency could also be explained by a lack of practitioner awareness or training, an excessive trivialization of this condition by some dentists, and/or the difficulty in accurately identifying and diagnosing OUBS, as it shares symptoms with other conditions like dry socket or osteonecrosis induced by certain medications or radiotherapy.

As the precise pathophysiology of OUBS remains unclear, and considering the scarcity of publications on OUBS, we think that it is legitimate to consider that any new clinical observation can be helpful in the understanding of this condition. In this context, we report a case of OUBS observed recently at the *Paris‐Dieb Dental* Clinics in Paris. The presentation is accompanied by some recommendations and is completed with a short discussion.

## 1 Case Presentation

**FIGURE 1:**
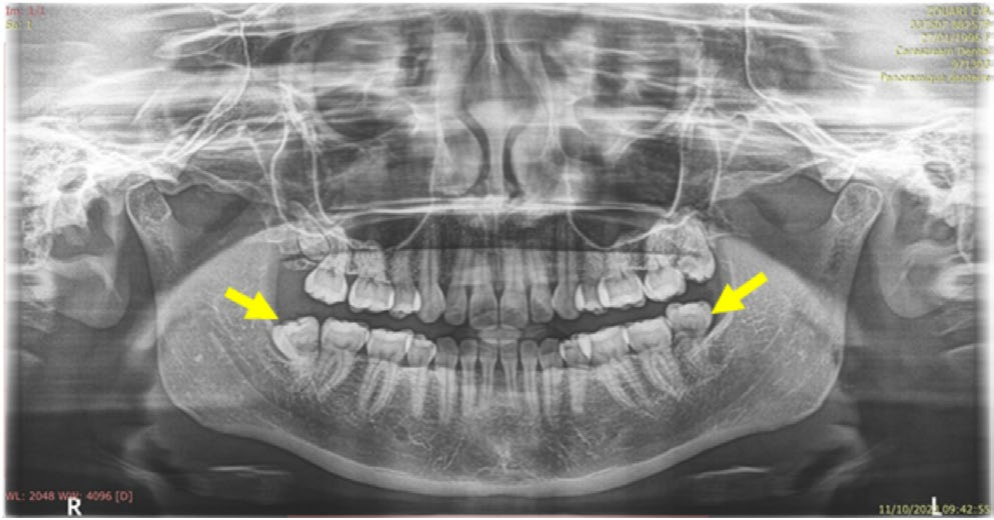
Orthopantomogram showing the positions of wisdom teeth 38 and 48 (yellow arrow).

Accompanied by a referral letter from her dentist, a 28‐year‐old woman presented at our dental surgery clinic on October 9th, 2023, with complaints of pericoronitis and a request for the extraction of her two mandibular wisdom teeth. As a heavy smoker (> 10 cigarettes a day), she had no other orofacial symptoms nor a history of predisposing systemic conditions (Figure 1).

After treating the pericoronitis locally and systemically, the vestibularly‐positioned wisdom teeth (48 first, then 38) were extracted on November 8th, 2023. Nevertheless, a small and healthy fragment of the apex of tooth 38 was accidentally fractured and then intentionally left in place and monitored, as recommended in the literature [5].

One week later, the patient reported intense pain at the site of tooth 48's extraction and was diagnosed with dry socket. Specific treatment was administered, including curettage of the extraction site, application of an Eugenol‐impregnated compress, and a course of systemic analgesics and antibiotics. The pain gradually subsided, and the extraction site healed within 3 weeks.

**FIGURE 2:**
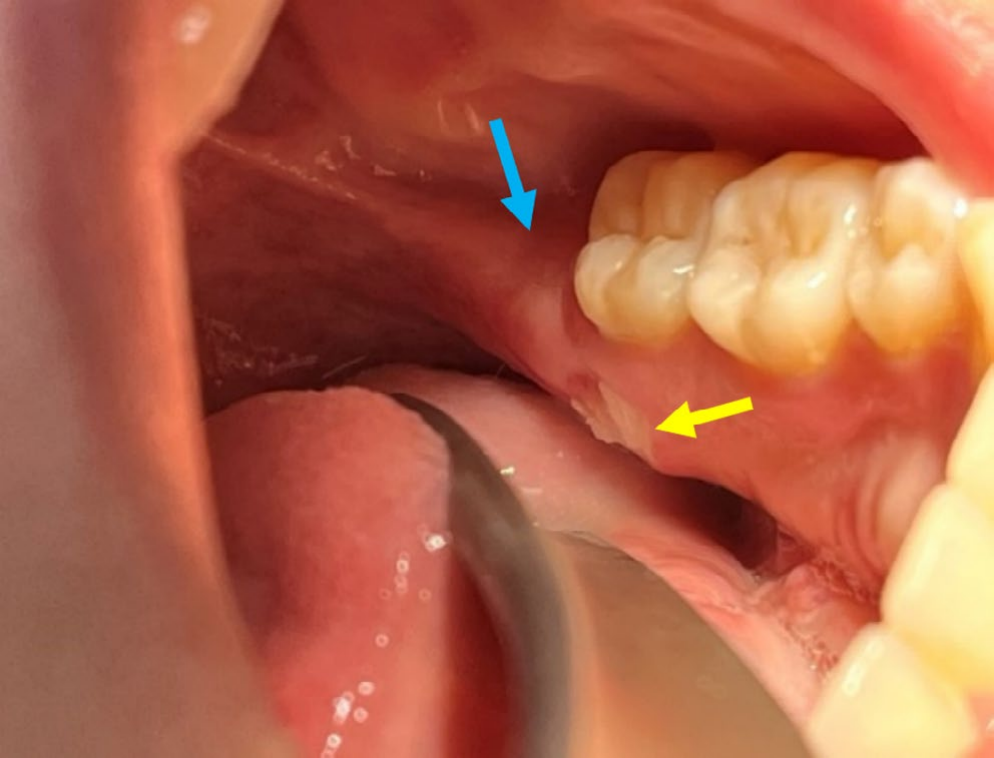
Ulcerative lesion (yellow arrow) on the left mylohyoid ridge lingually to tooth 37. Good healing of the extraction site of tooth 38 is visible (blue arrow).

Unfortunately, 3 months after the extraction, the patient reported discomfort and mild pain on the opposite side near the left mylohyoid ridge. On February 14th, 2024, a proper healing was observed at the extraction site of tooth 48, but a centimetric ulceration (1.8 × 1.4 cm) with an erythematous halo surrounding exposed bone with sequestration at the left mylohyoid ridge was observed (Figure 2). The margins of the surrounding soft tissues were painful both spontaneously and upon stimulation when the patient's tongue touched the lesion. X‐ray did not reveal any abnormalities.

The patient was prescribed antibiotics, and a follow‐up visit was scheduled for the following week. In the meantime, a detailed analysis of additional exam data and medical questionnaires was conducted.

### 1.1 Differential Diagnosis

During her next visit on February 21st, 2024, we observed that the antibiotics did not lead to any improvement, and, based on the medical and clinical observations and on a recent analysis of the scientific and medical literature, the differential diagnosis was established:

– Dry socket (characterized by intense pain and a seemingly empty cavity): Ruled out as the ulceration and bone exposure were not at the extraction site.
– Medication‐related osteonecrosis of the jaw: Excluded as there was no history of bisphosphonate or similar medication use.
– Aphthous ulcer: Excluded as this condition generally heals within 10–14 days and does not present exposed bone.
– Physical or chemical traumatic ulceration: Ruled out as there was no evidence of recent trauma or chemical exposure observed or reported by the patient.
– Neoplastic lesion: Excluded since radiography and clinical examination did not indicate signs of malignancy.
– OUBS: OUBS was the final diagnosis retained as it was the only one consistent with the clinical examination and the patient's reports.

### 1.2 Treatment

Consequently, the following combined protocol that relies on surgical removal of the sequestrum combined with antibiotics and the application of local disinfectants was implemented:

#### 1.2.1 Surgical Procedure

**FIGURE 3:**
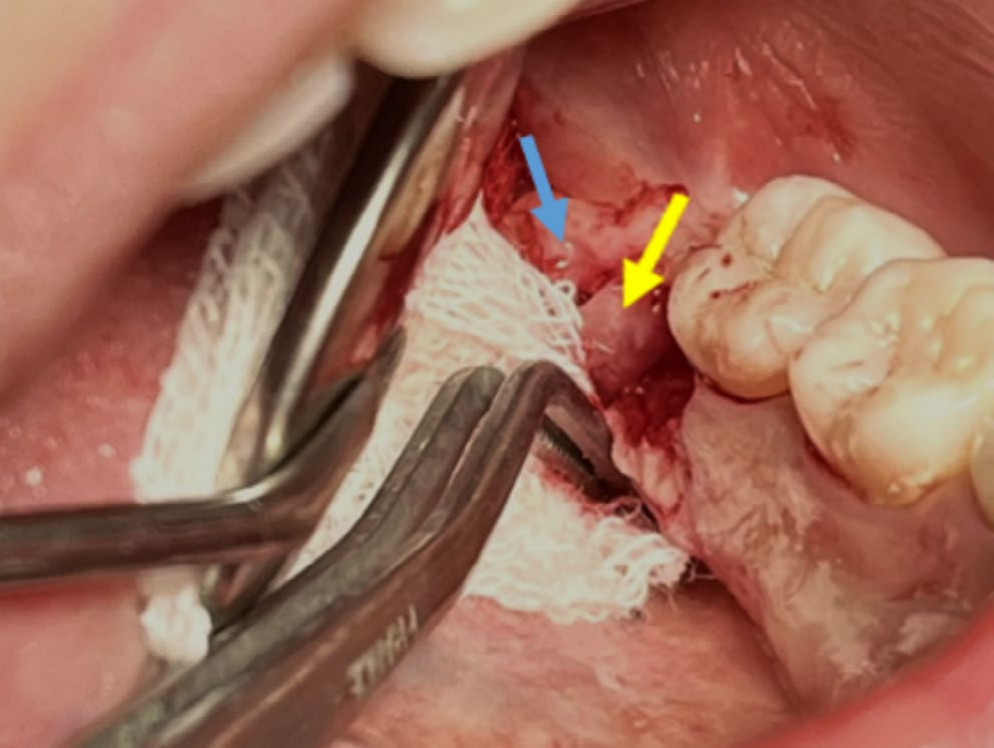
Two‐part flap to remove the sequestrum: The upper part (yellow arrow) covered the lingual limit of tooth 37, the lower part of the lesion (blue arrow) covered the posterior‐inferior limits of the lesion.

Surgery was carried out on February 27th, 2024. Under local anesthesia, the margins of the ulcer were gently elevated with a periosteal elevator, and the sequestered bone was removed with a surgical burr until reaching the vital bone layer (Figure 3). The freshly exposed bone was covered by the gingival flap repositioned lingually and sutured in place to promote healing. A Coe‐Pak surgical dressing covered the site.

#### 1.2.2 Pharmacological Procedure

The patient has been prescribed a course of antibiotics (amoxicillin 1 g twice daily and metronidazole 500 mg three times daily) for a week. Additionally, a mouthwash with chlorhexidine (0.12%) was prescribed three times a day for a week. To alleviate the pain, paracetamol 500–1000 mg with codeine 30–60 mg was prescribed to be used as needed. Only the usual postoperative instructions were given to the patient, including maintaining good oral hygiene with the help of antiseptic mouth rinses.

The chosen method was preferred due to its effectiveness in quickly removing necrotic bone and promoting faster healing, as evidenced by the rapid and complete healing within 30 days post‐surgery.

### 1.3 Follow Up

**FIGURE 4:**
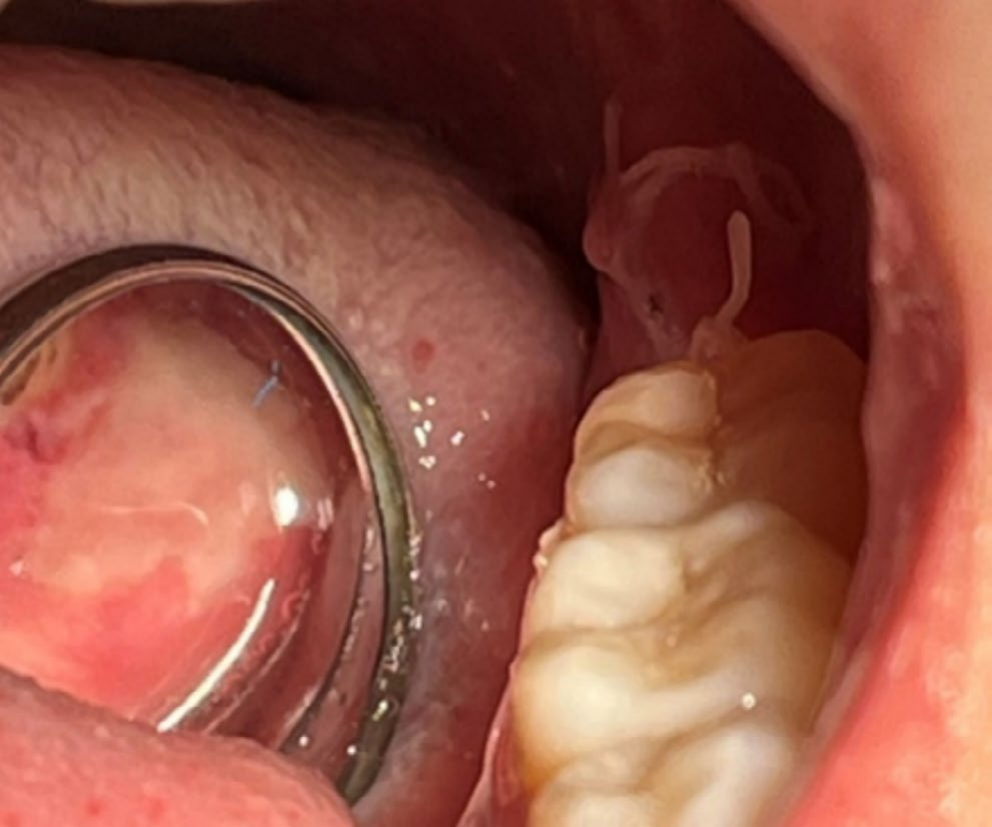
Initial epithelialization of the OUBS two days after the surgical intervention.

**FIGURE 5:**
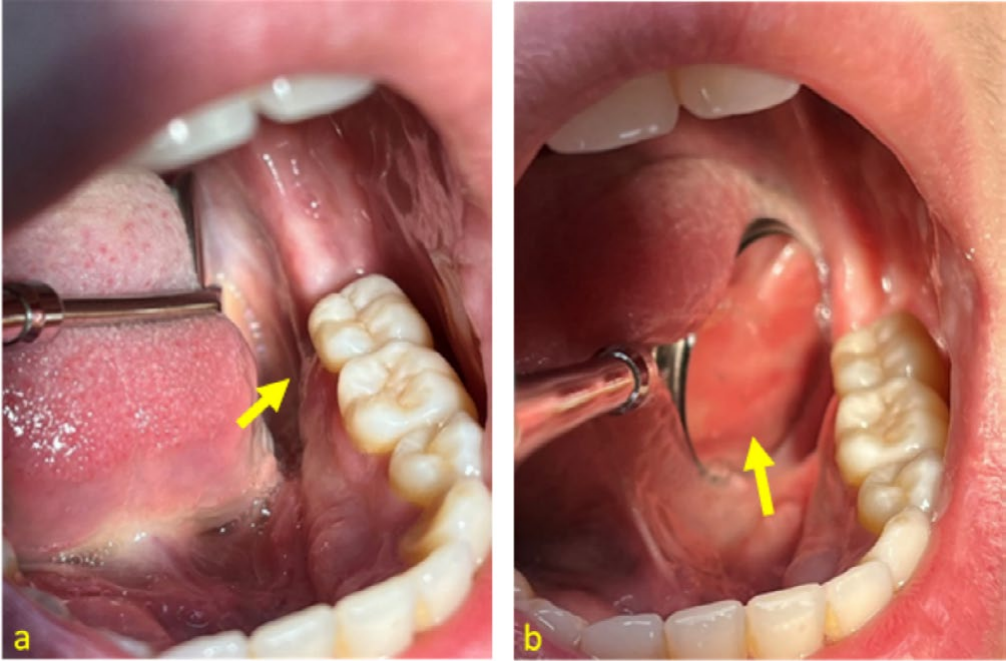
Direct view (a) and indirect view (b) of the OUBS lesion site showing complete healing four weeks post‐surgery.

The patient was re‐examined 2 days after surgery, during which a significant reduction in pain was reported. Upon clinical observation, the early stages of epithelialization were noted, indicating a positive initial healing response (Figure 4). These findings suggested that the combined surgical and pharmacological approach was effective in promoting tissue repair and managing postoperative symptoms. At clinical follow‐up at 30 days (March 29th, 2024), the site of the OUBS had completely healed (Figure 5), pain had completely disappeared, and the patient was totally satisfied.

## 2 Discussion

Our case report contributes to the limited but steadily growing body of literature on OUBS, providing valuable insights for further understanding and management of this condition.

### 2.1 About the Case Report

The presence of wisdom tooth 38 interacting with its opposing tooth 28 prevented direct traumatic injury to the already prominent lingual mandibular mucosa at the level of the mylohyoid ridge. We hypothesized that a few weeks after the extraction of tooth 38, our patient began chewing with her left teeth, resulting in a direct traumatic force on the mylohyoid ridge, inducing an acute mucosal lesion. Initially superficial, this lesion progressively affected the underlying poorly vascularized bone. Subsequently, partial or total necrosis set in, leading to bone sequestration.

Additionally, considering the fact that smoking can impair blood flow that is crucial for nutrient and oxygen delivery necessary for tissue repair and healing [6], it can be legitimate to consider it a contributing factor to the development of a dry socket at the site of tooth 48's extraction. Though we cannot ignore the potential impact of other systemic inflammatory or immunological factors such as anemia, AIDS, neutropenia, or hormonal imbalances, these factors were not investigated. Currently, no consensus on the management of OUBS exists [2, 3]. However, our experience led us to suggest the following a 3‐step management:

– *A preventive approach*: This step is decisive as it is essential to detect and eliminate (when possible) systemic risk factors such as smoking, obesity, and diabetes, and to control and correct any immune system deficiencies (e.g., AIDS, other immunodeficiency‐related diseases). It is also recommended to surgically remove tori and exostoses and to correct any insufficient inclination of the posterior mandibular alveolus by replacing missing teeth;
– *A therapeutic approach*: In our study, prescribing antibiotics alone was not sufficient to treat OUBS. This can be explained by the formation of a sequestrum at the lesion site, where the delivery of these drugs is hindered by a reduced microvascular supply, especially in cases of necrosis [4, 7, 8]. Consequently, surgically removing the sequestrum and applying a local disinfectant such as chlorhexidine followed by the administration of a targeted antibiotic regimen could be sufficient to ensure complete healing within 3 weeks; During the surgical procedure, specific measures were taken to mitigate potential risks of lingual nerve injury by employing careful dissection techniques and maintaining a safe distance from the nerve pathway.
– *A post‐surgery control*: Post‐surgical monitoring and regular follow‐up are essential to ensure complete healing and prevent recurrence.

It must be noted that other alternative treatments could be considered such as the long‐term use of antibiotics and antiseptic mouthwashes knowing that this less invasive approach would require more time to achieve healing and could fail if the sequestrum persists. For the future, it would not be absurd to consider the utilization of low‐level laser therapy that can support oral mucosa healing by reducing the exudative phase, stimulating tissue repair, and promoting the proliferation and differentiation of fibroblasts and myofibroblasts, and improving blood flow through angiogenesis, supporting revascularization and capillary growth, accelerating then healing and reducing patient discomfort. Tissue repair is further accelerated by the release of growth factors [9], not to mention that in some cases, a spontaneous healing (without surgery nor antibiotics) can be observed [2]. Regardless of the chosen management protocol, the patient should be re‐examined until full healing of the lesion that is generally achieved in less than 8 weeks after the removal of the sequestrum [2, 7, 10].

### 2.2 About OUBS

Oral ulcerations are common, but progression to osseous sequestration is extremely rare [11]. When it occurs, healthcare professionals should consider systemic predisposing factors such as the use of bisphosphonates, which can induce medication‐related osteonecrosis of the jaw, or radiotherapy, which can lead to osteoradionecrosis. Another condition similar to OUBS is alveolar osteitis or dry socket, which can also affect the maxillary bone but can be easily diagnosed by its exclusive development at the site of dental extraction [12]. Moreover, mucosal ulceration with osseous involvement might also indicate neoplasia that can be diagnosed clinically and confirmed through histopathology [13].

These four pathological situations potentially interfere with the definitive diagnosis of OUBS. In summary, to pinpoint the crucial aspect of the diagnosis, it is recommended to look for the lesion's topology and the absence of two systemic factors: medication use and radiotherapy.

The preferred site for OUBS is the posterior lingual mandibular mucosa along the mylohyoid ridge [2, 3, 4]. Thus, attention should be given to this site as it is also susceptible to osteonecrosis, with early stages appearing similar to OUBS, and clinicians must remind that whether it is OUBS or osteonecrosis, the posterior part of the mouth is generally difficult to clean with the tongue due to limited movement, promoting bacterial colonization of the lesion site and exacerbating the condition [4, 7, 8]. OUBS has also been observed in maxillary exostoses, mandibular tori, and at the posterior hard palate [14, 15, 16], even if other locations within the maxillary and mandibular bone must not be excluded [2]. In any case, these osseous topologies take the form of bone projections covered by thin, fragile mucosa, which suffer from poor blood supply, making them prone to infection. For example, the mylohyoid line has poor blood supply due to the distance from the lingual mandibular cortex to the intraosseous arteries [4]. Finally, it should also be noted that bilateral OUBS has been reported only twice in the literature: at the beginnings of the 1990s by Peters et al., who suggest that systemic factors (diabetes) might play a crucial role [4], and more recently by a Greek team in an elegant retrospective study [2].

Understand the topology of OUBS on oral bone prominences and its pathophysiology and clinical presentation are vital for accurate diagnosis. Heightened awareness and thorough differential diagnosis remain essential approaches for appropriate management and improved patient outcomes.

### 2.3 Limitation of the Study

This case report presents some limitations, including the absence of investigation of systemic factors such as immunological conditions that could contribute to OUBS. To our knowledge, the patient's smoking habit is the only identified risk factor that could interact with the development and healing of OUBS. Additionally, the potential influence of genetic factors cannot be ruled out. However, given the patient's full recovery, the absence of lesion recurrence during follow‐up visits, and the demonstrated effectiveness of the surgical treatment, we did not deem further examinations necessary in this instance.

Furthermore, the findings from our case may be relevant to similar patient populations, especially those with habits or living conditions that could impair oral healing, such as smokers and diabetics. Nevertheless, the unique nature of the case limits the ability to generalize our findings to patients with different medical situations. Consequently, further research involving more diverse populations is required to generalize these results.

## 3 Conclusion

While being under‐reported in the medical and scientific literature, OUBS is a pathological condition for which diagnosis requires a thorough clinical analysis, and that practitioners will statistically face in their professional practice. In our case, surgical treatment combined with antibiotics led to rapid healing. Our study highlights this pathology and its management through an original case report, assisting healthcare professionals in diagnosing and managing OUBS. Furthermore, we hope that this study will encourage practitioners to share their experiences of OUBS in case reports in the absence of a broader dissemination platform dedicated to this pathology.

## Author Contributions

**Marc Dieb:** conceptualization, investigation, methodology, supervision, writing – original draft, writing – review and editing. **Lea Dieb:** conceptualization, investigation, methodology, project administration, validation, visualization, writing – original draft, writing – review and editing. **Claude Robert:** conceptualization, investigation, methodology, supervision, writing – original draft, writing – review and editing.

## Ethics Statement

The authors declare that the procedures respected the recommendations of Article 13 of the GDPR and were followed according to the Helsinki Declaration of the World Medical Association.

## Consent

This article has been written according to the CARE recommendations for clinical cases. Written consent has been obtained from the patient for the publication of the data.

## Conflicts of Interest

The authors declare no conflicts of interest.

## Data Availability

Data openly available in a public repository that issues datasets with DOIs.
